# Improved bathymetry leads to >4000 new seamount predictions in the global ocean – but beware of phantom seamounts!

**DOI:** 10.14324/111.444/ucloe.000030

**Published:** 2021-12-22

**Authors:** Chris Yesson, Tom B. Letessier, Alex Nimmo-Smith, Phil Hosegood, Andrew S. Brierley, Marie Hardouin, Roland Proud

**Affiliations:** 1 Institute of Zoology, Zoological Society of London, Regent’s Park, London NW1 4RY, UK; 2 Pelagic Ecology Research Group, Scottish Oceans Institute, School of Biology, University of St Andrews, St Andrews, Fife KY16 9TS, UK; 3 School of Biological & Marine Science, University of Plymouth, Plymouth, Devon PL4 8AA, UK; 4 Imperial College London, Silwood Park, Ascot, Berkshire SL5 7PY, UK

**Keywords:** seamounts, knolls, bathymetry, environmental science

## Abstract

Seamounts are important marine habitats that are hotspots of species diversity. Relatively shallow peaks, increased productivity and offshore locations make seamounts vulnerable to human impact and difficult to protect. Present estimates of seamount numbers vary from anywhere between 10,000 to more than 60,000. Seamount locations can be estimated by extracting large, cone-like features from bathymetry grids (based on criteria of size and shape). These predicted seamounts are a useful reference for marine researchers and can help direct exploratory surveys. However, these predictions are dependent on the quality of the surveys underpinning the bathymetry. Historically, quality has been patchy, but is improving as mapping efforts step up towards the target of complete seabed coverage by 2030. This study presents an update of seamount predictions based on SRTM30 PLUS global bathymetry version 11 and examines a potential source of error in these predictions. This update was prompted by a seamount survey in the British Indian Ocean Territory in 2016, where locations of two putative seamounts were visited. These ‘seamounts’ were targeted based on previous predictions, but these features were not detected during echosounder surveys. An examination of UK hydrographic office navigational (Admiralty) charts for the area showed that the summits of these putative features had soundings reporting ‘no bottom detected at this depth’ where ‘this depth’ was similar to the seabed reported from the bathymetry grids: we suspect that these features likely resulted from an initial misreading of the charts. We show that 15 ‘phantom seamount’ features, derived from a misinterpretation of no bottom sounding data, persist in current global bathymetry grids and updated seamount predictions. Overall, we predict 37,889 seamounts, an increase of 4437 from the previous predictions derived from an older global bathymetry grid (SRTM30 PLUS v6). This increase is due to greater detail in newer bathymetry grids as acoustic mapping of the seabed expands. The new seamount predictions are available at https://doi.pangaea.de/10.1594/PANGAEA.921688.

## Introduction

Seamounts are ‘undersea mountains’, and although many definitions of this term have been used, they are commonly described as conical features that rise more than 1000 m above the surrounding seabed [1]. Seamounts are important marine habitats, they provide a pathway for localised production [2], often increasing surrounding biomass and species diversity [3], they can be hotspots of predator biodiversity in the open ocean [4], home to habitat-engineering species such as cold water corals [5], important spawning grounds [6], and even act as refugia from ocean acidification for carbon-calcifying species [7].

The increased productivity associated with seamounts makes them attractive targets for fishing. Fishing gear can cause long-lasting damage to habitat forming organisms associated with some seamounts [8]. Other threats to seamounts include deep-sea mining and climate change, with shallower, more accessible seamounts at greater threat. Protection of seamount habitats is a priority for marine conservation [9], but our knowledge on these habitats remains limited, with estimates of only 0.4–4% of seamounts having been directly surveyed [10].

Direct surveys require significant investment of resources and planning, and fundamental to this is identification of locations of interest for the survey. However, we do not yet know how exactly many seamounts there are, with estimates ranging from the tens to hundreds of thousands [11]. This has led to the publication of many predictive maps and databases of potential seamount locations, commonly based on pattern recognition of underlying bathymetry data [11–13], but also using satellite altimetry to detect larger features [14,15].

Seamount predictive maps are dependent on the underlying data to extract features. Global bathymetry grids such as GEBCO (General Bathymetric Chart of the Oceans) – [16] and SRTM (Shuttle Radar Topography Mission [17]) are models based on a combination of soundings (i.e., high resolution acoustic surveys) and satellite altimetry (lower resolution data from satellite sensors). Satellite altimetry provides global coverage and is the foundation of bathymetry models, but these sensors cannot determine small features (i.e., seamounts under 1.5 km height [14]). Acoustic surveys generate data best suited for determining seabed depth and these are utilised to constrain models used to create bathymetry grids [17]. Despite global efforts to improve coverage, such as the Nippon Foundation-GEBCO challenge to survey the ocean floor across the globe by 2030 [18], soundings in the latest bathymetry grids are limited to a small proportion of the ocean, and the majority of bathymetry grid data is derived from the underlying model rather than acoustically surveyed. For example, only 18% of current GEBCO grid cells (each 30 × 30 arc seconds ≈ 1 × 1 km at the equator) are directly supported by acoustic surveys [16]. As sounding data is limited, it is valuable to make use of all available data. Historical soundings based on weighted lines have been extracted from nautical charts to expand the data available [17].

This study describes issues with seamount predictions stemming from the use of historical sounding records, based on the findings of a seamount survey in the Indian Ocean. It presents an update of previous seamount predictions and examines whether this erroneous use of historical data persists.

## BIOT seamount survey

**Figure 1:**
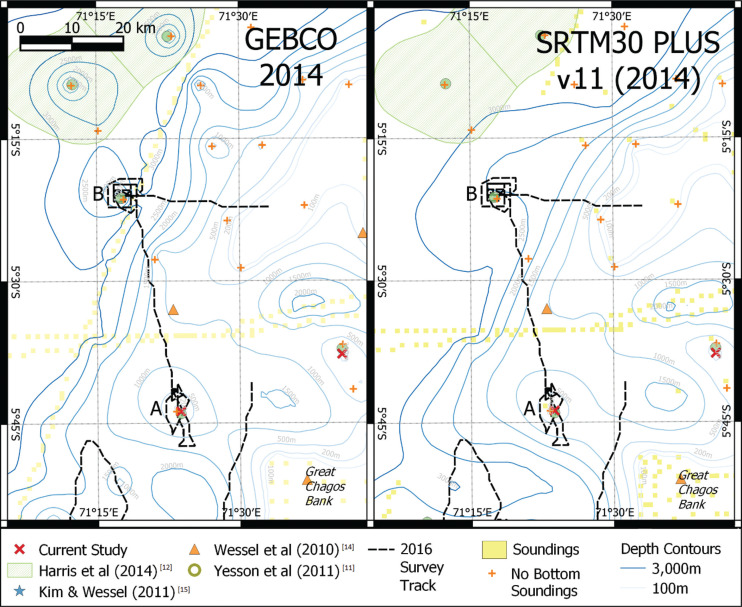
Location of survey conducted in 2016. Left shows depth contours based on the 2014 GEBCO bathymetry grid, right shows depth contours derived from SRTM30 PLUS v11. Both grids indicate the presence of a conical seamount (A) c.20 km NW of the Great Chagos Bank. No feature was detected by the 2016 survey. Around 40 km north of this, is another predicted seamount (B), again not detected on the 2016 survey. Feature B is predicted by the GEBCO grid but is not shown in the SRTM30 PLUS grid (although present in previous versions). Map projection UTM zone 43 south (epsg:32743).

**Figure 2:**
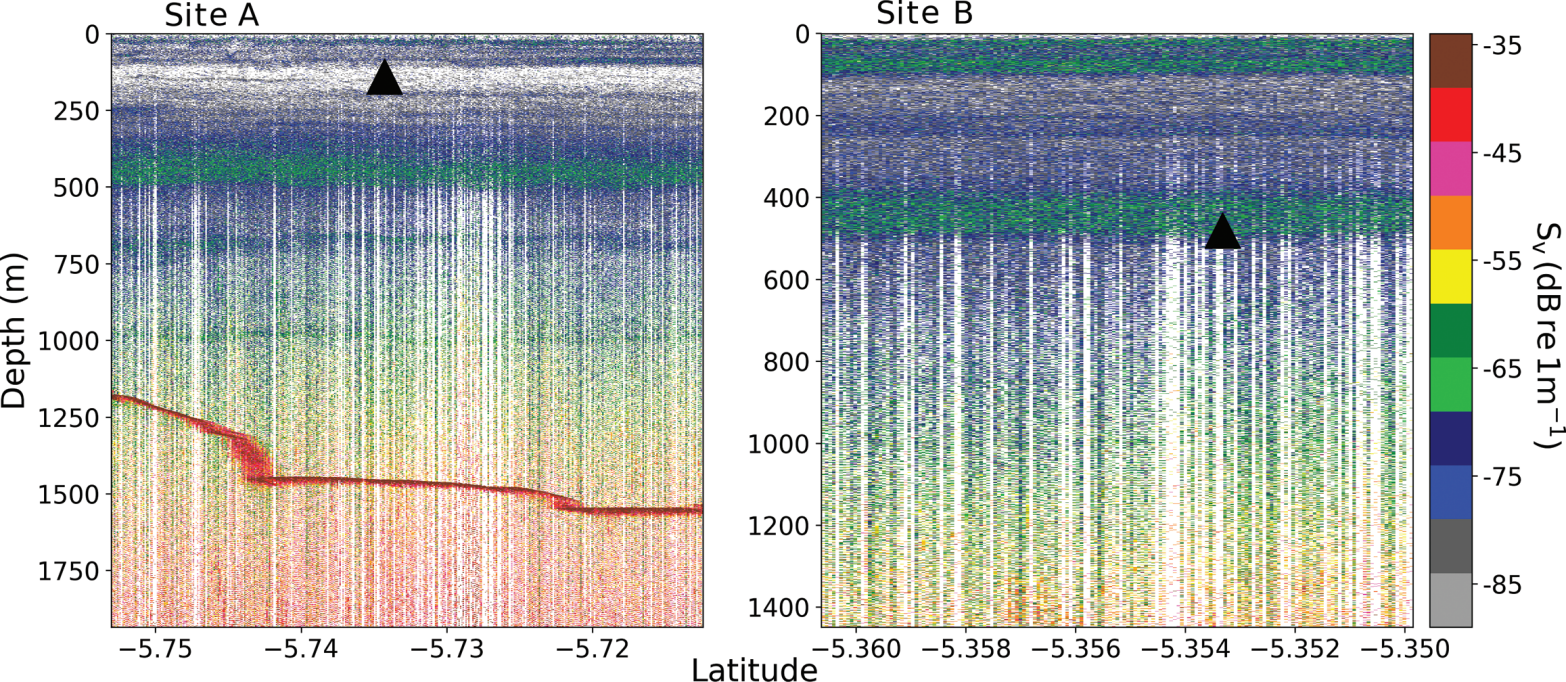
Latitudinal transects across apparent positions of the two ‘phantom seamounts’. Black triangles are overlayed at the position and summit depth of the predicted seamounts. Colormap is volume backscattering strength (Sv). A deep scattering layer was observed at c.450 m for both sites. Seabed was observed at site A c.1500 m (red line). No seabed was detected for site B (i.e., seabed is deeper than the limit of the sensor).

The British Indian Ocean Territory (BIOT) is a region of the Indian Ocean encompassing a variety of undersea features, including the flat shallow banks of the Chagos Archipelago, and the high slopes of the Chagos–Laccadive ridge, and depths beyond 5000 m [19]. The area could be home to as many as 86 seamounts, based on estimates from an automated seamount-recognition algorithm applied to version 6 of the SRTM30 PLUS global bathymetry grid [11]. Two of these predicted seamounts, clearly discernible on the latest bathymetry grids, were targeted during a 2016 multidisciplinary survey around the Chagos Archipelago [20]. The seamount section of the survey moved around the Great Chagos bank spanning c.5–7°S and 71–73°E, between 5 and 24 February. Two seamounts of interest were ID 4050548 (latitude −5.354, longitude 71.292, summit depth 481 m) and ID 4060551 (lat. −5.733, long. 71.396, depth 141 m) from Yesson et al. [11]. The survey sought to visit these features for the purpose of establishing baseline monitoring sites for mobile oceanic predators [21]. Seamounts in BIOT have previously been shown to be important locations of bio-physical coupling between reef and pelagic ecosystems, and may therefore support elevated numbers of predators [2,22,23]. Acoustic data were collected using a Simrad (Bergen, Norway) EK60 echosounder operating at 38 kHz with a pulse length of 1.024 ms and ping rate of 2 s. At these settings, the seabed was detectable up to 1500 m below the surface. Seabed was detected at around this depth for seamount A (predicted depth 183 m), but no seabed was detected around the area of seamount B (predicted depth 491 m) despite circling (up to 5 km) around the supposed summits (Figs 1 and 2). We note that the source of the reading that accounts for seamount B was a digital nautical chart from the National Geospatial Agency and this erroneous point is removed from construction of more recent bathymetry grids (D. Sandwell, pers. comm.).

An examination of the admiralty chart for the region provided some insight. Soundings on charts are recorded by displaying the depth reading over the location. A different class of sounding is also recorded. Soundings where no bottom was recorded are annotated with 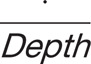 at the location of the sounding. These soundings are typically old, prior to the 19th century, dating from when soundings were conducted using handheld, weighted, lead lines, before the widespread use of sounding machines. It is easy to mistake these as bottom soundings, and this appears to be the root cause of the ‘phantom seamounts’. For site A (Fig. 1) there is a sounding in the chart at the summit of the mound seen on the bathymetry grids. The chart reports no bottom recorded at 183 m, while the GEBCO depth at this cell is 179 m and SRTM30 PLUS depth is 183 m.

However, the SRTM30 PLUS grid at site B does not show a seamount-like feature, in contrast to GEBCO, which shows an isolated point of markedly higher elevation, which is interpreted as a conical seamount-like peak by seamount detection algorithms. It is noted that previous versions of the SRTM30 PLUS grid showed a seamount-like feature at this location. The version history reports the removal of isolated and outlier ‘bad pings’ prior to the construction of version 11. The revision of SRTM has removed other seamount-like features from the revised bathymetry grid [i.e., northwest (NW) corner of Fig. 1]. It is apparent that bathymetry grids such as GEBCO and SRTM30 PLUS have mistakenly used these ‘no seabed detected’ observations as soundings indicating seabed depth, and in regions with sparse sounding data, these spatially isolated erroneously interpreted records are sufficient to create a local maximum that creates the appearance of a seamount in the final bathymetry grid.

This study aims to update the Yesson et al. [11] seamount predictions using the latest available bathymetry and assess the impact of no bottom sounding data on the prediction of seamounts.

## Methods

Version 11 of the Shuttle Radar Topography Mission ‘SRTM30 PLUS’ global bathymetry ([17] – version 11 released 2014) was used to update the seamount prediction estimates of Yesson et al. [11]. The prediction algorithm of Yesson et al. [11], which identifies seamounts as cone-shaped features rising more than 1000 m above the surrounding seabed, was run on SRTM30 PLUS v11, creating a new set of seamount predictions based solely on the new bathymetry.

New seamount predictions were compared with the previous dataset ([11] – henceforward the ‘old’ dataset). Seamounts were defined as present in the old dataset if the base of a seamount in the new dataset spatially overlapped with a seamount summit in the old seamount dataset (i.e., both datasets have a predicted seamount in approximately the same location). Seamount bases are the area covered by the ‘cone’ of the seamount, and are delimited by 8 radii 45° apart, radiating from the seamount summit point, that extend outwards from this point until the downward slope levels off, up to a maximum distance of 20 km from the summit (thus the maximum base area is ∼1131 km^2^). These seamount bases can, and often do, encompass multiple seamount peaks in both the old and new datasets, but a new seamount has to overlap with just one seamount in the old dataset to count as being a consistent prediction.

A dataset of no bottom sounding observations was provided by OceanWise Ltd (Alton, UK), from a dataset of depth readings from digitised admiralty charts. These data include 1009 observations from charts covering the majority of the Atlantic and East Pacific, but with little data from the Southwest Indian Ocean and West Pacific. The depth readings of no bottom soundings that were spatially located within seamount bases were compared with the summit depths, seamounts with peak-depth similar to no bottom sounding depths (+/−50 m) were regarded as potential ‘phantom seamounts’.

## Results

**Figure 3:**
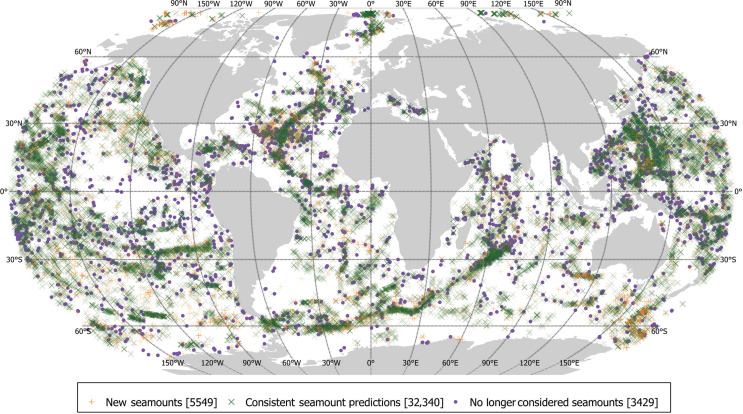
Map of predicted seamounts. New Seamounts are those in the new dataset that are not found in the Yesson et al. [11] dataset. ‘Consistent predictions’ are new predictions that spatially overlap with the old predictions of Yesson et al. [11], while those seamounts present in Yesson et al. [11] but with no overlapping feature in the updated dataset are classed ‘no longer considered seamounts’. Robinson map projection (EPSG:54030). Lat/Long grid lines at 30° intervals.

The updated seamount predictions based on the SRTM30 PLUS v11 bathymetry gives a total of is 37,889 seamounts. A map of these is presented in Fig. 3. There are 32,340 ‘consistent’ seamounts in the new dataset that overlap with predictions from Yesson et al. [11] and 5549 ‘new seamounts’ (15%) that do not overlap with old predictions. Conversely, there are 3429 seamount predictions in the old dataset (=10% of old seamount predictions) that do not overlap with the seamount bases of the new dataset.

**Figure 4:**
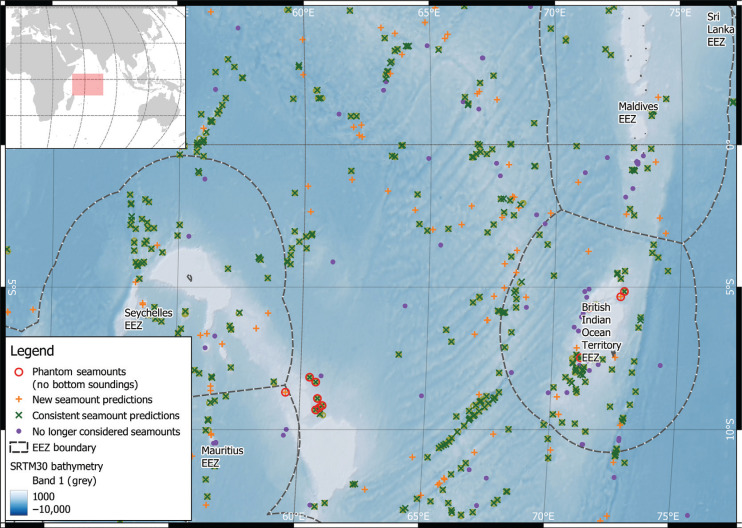
Focus on seamounts of NW Indian Ocean. Most of the 15 predicted seamounts based on no bottom soundings are in the Indian Ocean. EEZ are exclusion economic zones (boundary of national jurisdiction – source https://www.marineregions.org/). Robinson map projection (EPSG:54030). Lat/long lines shown for reference.

There are only 15 seamounts in the new dataset that fit a ‘phantom seamount’ profile (i.e., near a no bottom sounding record with the seamount peak of similar depth to the sounding record), these are presented in Table 1. In contrast there are 14 seamounts from the ‘old’ 2011 dataset that fit this pattern. These ‘phantom seamounts’ are focused in the Indian Ocean (12/14 from 2011 data and 12/15 from the updated dataset), with four potential ‘phantom seamounts’ around Chagos Bank and six from the southern Mascarene Plateau (Fig. 4).

**Table 1.** List of ‘phantom seamounts’ where inferred seamounts appear coincide with sites of no bottom soundings

Peak ID	Depth (m)	Height (m)	Longitude	Latitude
4509328	52	1732	59.42083	−8.68750
4523965	2	2015	60.79583	−9.22917
4525766	2	2051	60.65417	−9.30417
4515124	65	2114	60.70417	−8.90417
4475075	304	2267	71.12917	−7.47917
4408881	191	2354	72.78750	−5.15417
4521899	2	2409	60.90417	−9.15417
4414134	135	2481	72.64583	−5.33750
844462	166	2712	71.39583	−5.72917
3736711	2	2802	−65.93750	17.80417
888460	2	3068	43.92083	−12.38750
4495055	54	3676	60.36250	−8.16250
699884	133	3752	144.38750	12.77917
4499613	85	3762	60.61250	−8.32917
4264476	17	6361	−159.97917	−0.37917

**Figure 5:**
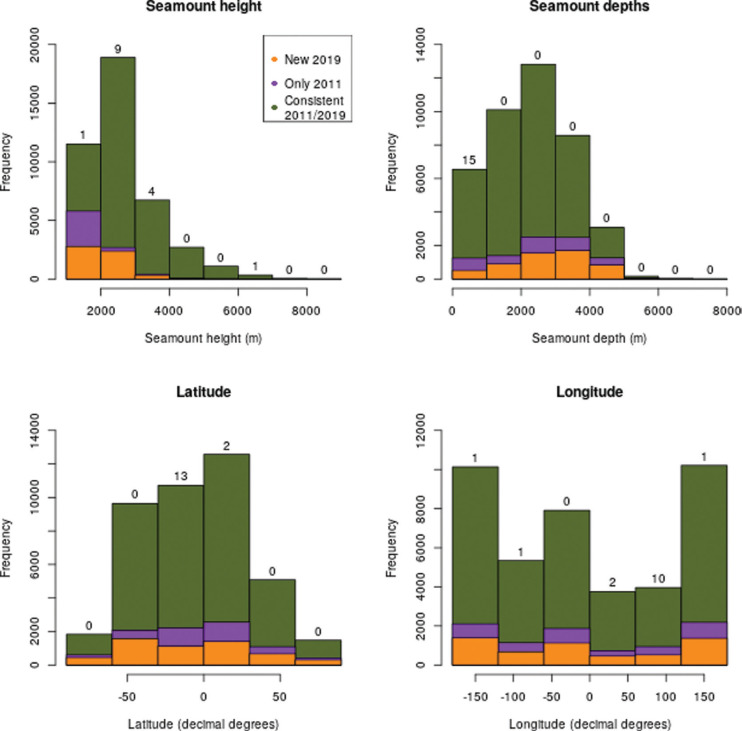
Histograms showing distribution of seamounts by seamount height (top left), depth of seamount summit (top right), and geographic location of seamount (latitude – bottom left, longitude – bottom right). Numbers above the bars show the count of ‘phantom seamounts’ in the relevant grouping.

The ‘phantom seamounts’ are all in shallow water (summit depth <1500 m) and most are in the southern hemisphere (Fig. 5). The majority of seamounts are at the smaller end of the size distribution and typically found at 2000–3000 m depth (Fig. 5). However, the ‘new’ seamounts from the 2019 data are overrepresented in the smaller and deeper categories, while the seamounts only seen in the 2011 dataset are greatly focused on the smallest size class.

## Discussion

The 37,889 seamounts predicted from the latest SRTM30 PLUS bathymetry represents an increase in number (4437 = 13%) of seamounts predicted from the previous study (*n* = 33,452). The revised predictions are higher than other predictions that post-date Yesson et al. [11], such as 24,643 seamounts in the Kim and Wessel [15] dataset and 10,234 of Harris et al. [12], but are still lower than some other predictions, for example, 68,669 of Costello et al. [24]. It is worth noting that each of these studies uses different ways of detecting seamounts, for example, Harris et al. [12] have a stricter definition of seamount that excludes features along ridges, while the methodology used in this study (from Yesson et al. [11]) employs a distance-based filtering of adjacent features.

Regardless of the methodology used, it is important to keep prediction datasets up to date with the latest bathymetry grids. We note that a global 15 second bathymetry grid is available (SRTM15+ v2.1 [25]), and that this greater detail may assist with seamount identification, although may require adjustment of the current methodology to fully utilise [12]. We expect the expansion of multibeam echosounder data [18] to allow the detection of smaller (<1.5 km) features in regions where previously bathymetry grids relied on only coarse resolution satellite-derived data, which is why authors have extrapolated their ‘detected’ seamount numbers to higher global estimates (e.g. [15] detect 24,643 seamounts, but extrapolate this to a global total of 40,000–55,000). This pattern of increased seamount detection as more acoustic data becomes available fits our observation and we note that the majority of ‘new’ seamounts are in the smaller, deeper size and depth categories, which is consistent with greater acoustic data giving more detailed resolution. We also note that these totals are really counts of seamount peaks, some of which may be linked together into seamount chains which could be regarded as a single feature. This potential double-counting may become more prevalent as these features are mapped in greater detail and smaller peaks on larger structures are identified. It was to address this issue that Yesson et al. [11] introduced an optional filter to remove spatially adjacent features, and we recommend always examining the filtered and unfiltered predictions with this in mind.

However, there is a competing pressure that may lead to a reduction of seamount numbers, as isolated no bottom soundings or erroneous readings, such as those identified in this study, are removed from bathymetry grid construction, so features defined by these mistakes should be removed as underlying grids are improved [16,17]. It is imperative that our predictions are as accurate as possible, as every erroneously identified feature could prove costly in terms of the investment required to conduct a research cruise to a ‘phantom seamount’ or the negative effects of taking protection measures for non-existent features. Fortunately, the scale of the problem directly identified in this study appears to be small and will likely reduce as methods improve and primary data collection expands. However, not all of these no bottom sounding records have been removed and there may be other causes of error not currently identified.

Finally, although these predictions are based on a global bathymetry grid, we note that seamount predictions based on the lat.–long. bathymetry grid perform poorly at high latitudes where there is a large spatial distortion. Seamount predictions for Arctic and Antarctic regions should be remade based on polar specific grids such as the International Bathymetric Chart of the Arctic Ocean (IBCAO [26]).

## Conclusion

Bathymetry grids are continually improving [18], whether that is from new multibeam acquisition, such as that collected during the search for Malaysian Airlines flight MH370 [27], or improved satellite gravity data [28]. However, these bathymetry grids still rely on sparse sounding data for many regions, and thus have the capacity to mislead if invalid historical weighted line measurements are used in the construction of bathymetric models as isolated falsely interpreted records can lead to the appearance of ‘phantom seamounts’. Despite advances in data acquisition, modelling and prediction methods, these data will continue to contain errors. Therefore, it is important that we use all the information available, including multiple seamount predictions, multiple bathymetry models and printed charts to assess potential seamount distributions, particularly when planning surveys to unsampled seamounts, or in the arena of conservation planning, where seamount distributions can be used as proxies for endangered predator distributions [29].

## Data Availability

Data availability: The datasets generated during and/or analysed during the current study are available in the repository: https://doi.pangaea.de/10.1594/PANGAEA.921688.
